# Selenite Inhibits Notch Signaling in Cells and Mice

**DOI:** 10.3390/ijms22052518

**Published:** 2021-03-03

**Authors:** Michael Powers, Liu Liu, Dane Deemer, Selina Chen, Aaron Scholl, Masafumi Yoshinaga, Zijuan Liu

**Affiliations:** 1 Department of Biological Sciences, Oakland University, Rochester, MI 48309, USA; michaelpowers@oakland.edu (M.P.); liu316msc@gmail.com (L.L.); deemergd92@gmail.com (D.D.); selinachen@oakland.edu (S.C.); ajscholl@oakland.edu (A.S.); 2 Department of Cellular Biology and Pharmacology, Herbert Wertheim College of Medicine, Florida International University, Miami, FL 33199, USA; myoshina@fiu.edu

**Keywords:** selenium, selenite, Notch, RNA-seq, inhibition, cancer

## Abstract

Selenium is an essential micronutrient with a wide range of biological effects in mammals. The inorganic form of selenium, selenite, is supplemented to relieve individuals with selenium deficiency and to alleviate associated symptoms. Additionally, physiological and supranutritional selenite have shown selectively higher affinity and toxicity towards cancer cells, highlighting their potential to serve as chemotherapeutic agents or adjuvants. At varying doses, selenite extensively regulates cellular signaling and modulates many cellular processes. In this study, we report the identification of Delta–Notch signaling as a previously uncharacterized selenite inhibited target. Our transcriptomic results in selenite treated primary mouse hepatocytes revealed that the transcription of Notch1, Notch2, Hes1, Maml1, Furin and c-Myc were all decreased following selenite treatment. We further showed that selenite can inhibit Notch1 expression in cultured MCF7 breast adenocarcinoma cells and HEPG2 liver carcinoma cells. In mice acutely treated with 2.5 mg/kg selenite via intraperitoneal injection, we found that Notch1 expression was drastically lowered in liver and kidney tissues by 90% and 70%, respectively. Combined, these results support selenite as a novel inhibitor of Notch signaling, and a plausible mechanism of inhibition has been proposed. This discovery highlights the potential value of selenite applied in a pathological context where Notch is a key drug target in diseases such as cancer, fibrosis, and neurodegenerative disorders.

## 1. Introduction

Selenium (Se) is an essential trace element found in all mammals and is vital for the proper functioning of selenoproteins, a unique group of proteins containing the rare but required amino acid, selenocysteine. Many selenoproteins including glutathione peroxidase (GPX) and thioredoxin reductase (TR) are antioxidants responsible for detoxifying reactive superoxide species (ROS) [1].

Selenium exists in both organic and inorganic forms with distinct cellular functions and toxicity. The organoselenium compounds such as methylselenocysteine and selenomethionine have been extensively studied and shown to be potent inhibitors of angiogenesis and cancers at supra-nutritional doses [2,3]. One form of inorganic selenium, anionic selenite (H_2_SeO_3_, dominantly HSeO_3_^−^ in physiological pH), has been clinically adopted to treat Keshan disease. Keshan’s is an endemic disease caused by dietary selenium deficiency, ultimately leading to cardiovascular atrophy if left untreated [4]. Selenite supplemented via diet effectively rescues the symptoms observed in Keshan disease, indicating selenite’s potential to serve a wide range of biological functions.

Selenite is known to have a higher affinity towards cancer cells [5]. This could be explained by the elevated levels of glutathione (GSH) in cancer cell microenvironments [6,7,8]. GSH facilitates selenite reduction to a free-permeable form, selenide (HSe^−^) [9]. Selenide, as a metabolite, is unstable and extremely toxic, increasing the production of reactive oxidative species (ROS) within cells. The ROS are then responsible for causing cellular damage and increasing lipid peroxidation; ultimately, triggering one of many cell death pathways [10]. The elevated rate of selenite uptake in cancer cells explains the vulnerability of cancer tissue to selenite and highlights the role of selenite in cancer therapy [11]. After reduction by GSH, selenite can be transported via the cysteine exchanger, Xc- (encoded by *Slc7a11*) [12]. Our lab has since demonstrated that selenite can also be directly transported into cells via a Zn^2+^ and bicarbonate (HCO_3_^−^) cotransporter, ZIP8, encoded by *Slc39a8* [13]. We believe that both mechanisms commonly exist and are key to the influx of selenite in cancerous tissues. On the other hand, selenite at higher concentration has genotoxicity, therefore the dose is critical in selenite treatment [14]. Clinically, selenite has been tested in the treatment of patients with carcinoma (Phase I: The SECAR) and has proven to be safe and well tolerated as a chemotherapy adjuvant [15].

In addition to its application as an anti-cancer agent, selenite effectively minimizes fibrogenesis in renal, hepatic, and pulmonary diseases as well as cystic fibrosis due to its inhibitory interactions with zinc metalloproteinases [16]. Selenite treatment likely increases the activity of selenoproteins crucial for maintaining ER stress while negatively affecting metalloproteinases such as matrix metalloproteinases (MMPs) and adamalysins or disintegrin metalloproteinases (ADAMs), necessary for fibrogenesis [17].

Selenite behaves in a dose-dependent manner. At low or physiological concentrations of selenite, the anti-apoptotic pathway is activated resulting in suppression of the apoptotic pathway, ultimately leading to selenite-induced proliferation [18]. At higher doses, with concentrations depending on the cell type, selenite can induce apoptosis by modulating reactive oxidative stress pathways [5,19,20].

In this study, we discovered a previously uncharacterized selenite inhibitory target, Delta–Notch signaling. This inhibition was initially identified using transcriptomic assays in primary cultured hepatocytes under Se-deficient conditions. This relationship was further investigated through sequential experiments in established cancer cell lines and intact animals, which may suggest that such inhibition is ubiquitous across cells and tissues.

Notch signaling is a fundamental and highly conserved pathway for determining many aspects involved in cell fate [21]. The Notch family contains four members, Notch 1–4, expressed in nearly all mammalian tissue. Studies containing gain or loss of function Notch 1–4 are associated with a wide range of diseases and disorders, including heart disease, multi-organ fibrosis, neurological disorders, and countless others [21]. Faulty Notch signaling has been implicated in the progression of various cancers, and it is suspected that Notch signaling may emerge as a plausible drug target. However, it remains unclear whether the activation or inhibition of Notch acts better as an anti-tumor agent due to the variation of cancer being treated [22]. Notch dysregulation has been found to play a deciding role in fibrotic disease progression [17] and is responsible for the differentiation of pro-fibrotic myofibroblasts using the TGF-β1-Smad3 pathways [16,17]. Epithelial–mesenchymal transition (EMT) has also been linked to Notch-related fibrogenesis [23].

Notch signaling has been found to participate in maintaining neural stemness and is implicated in both protective and harmful roles in neurodegenerative diseases such as schizophrenia, Alzheimer’s disease (AD), and multiple sclerosis (MS) [24]. The γ-secretase complex, necessary for Notch activation, was identified as a key enzyme in the pathological progression of Alzheimer’s disease (AD). As a result, the γ-secretase complex was proposed as a plausible drug target for the treatment of neurological diseases for both schizophrenia and AD [24]. Despite the promising preliminary findings, the connection between Notch signaling and complicated neurological diseases calls for more data.

Seeing as this pathway has been established in a variety of pathologies, searching for Notch inhibitors/activators is critical for the prevention and treatment of these complex diseases. As we report here, selenite, a clinically applied reagent, was identified as a potent inhibitor for Notch signaling in primary isolated hepatocytes, established cell lines (MCF7 and HEPG2), and in both the liver and kidneys of mice. Identification of selenite in the regulation of Notch signaling enhances our understanding of its role in clinical applications.

## 2. Materials and Methods

### 2.1. Cell Culture

Primary hepatocytes were isolated from an eight-week-old male mouse line, ZIP8-liver specific knockout (ZIP8-LSKO). This line originated from commercial C57BL/6 with *Slc39a8* floxed (Taconic, Germantown, NY, USA; model 11296) and was interbred with albumin transgenic mice (Alb-Cre^+/+^ Jackson lab). The mice used to isolate hepatocytes were harem bred; two eight-week-old LSKO females and one eight-week-old ZIP8 floxed male were placed together and observed until birth. Hepatocytes from LSKO mice have a reported 60% decrease in ZIP8 activity, a known selenite transporter [13,25]. The mice were randomly selected and individually placed in a CO_2_ gas chamber for euthanasia according to IACUC protocol. When mice fell unconscious, bilateral pneumothorax was verified to ensure mouse death before any further procedures ensued. We performed a two-step collagenase perfusion method as previously described [26]. A slow 1 mL/min perfusion at 37 °C was adopted before increasing the perfusion rate to 5 mL/min and cutting the IVC to allow drainage. Following perfusion, the livers were excised and gently pulled apart using sterilized forceps in a 0.1% collagenase solution (Collagenase Type 4, MP Biomedicals, Solon, OH, USA; # SCR103) containing DMEM/F12 (ThermoFisher, Waltham MA; #A4192002). The suspensions were filtered through a 74 μm stainless steel filter. Primary liver cell suspensions were centrifuged at 50× *g* and the cell pellets were collected. Cells were stained 1:1 with 20 μL of 0.4% trypan blue (Millipore Sigma, Burlington, MA, USA; #T8154-20ML) and quantified using the LUNA™ Automated Cell Counter (Logos Biosystems; Annandale, VA, USA). A total of 4.0 × 10^5^ viable hepatocytes were seeded per well on collagen-coated (Collagen I, rat-tail, ThermoFisher, Waltham, MA, USA; #A1048301) 6-well plates (USAScientific, Ocala, FL, USA; #CC7682-7506). Hepatocytes were cultured in serum-free basal medium (DMEM/F-12, 10 μM dexamethasone (Sigma-Aldrich, St. Louis, MO, USA; D4902) and 1% penicillin/streptomycin (ThermoFisher, Waltham, MA, USA; #15140122)) overnight in standard incubator conditions; 5% CO_2_ at 37 °C. For the selenite treatment, primary hepatocytes were incubated an additional 24 h in serum-free basal medium, as described above, with a final added concentration of 2 μM sodium selenite (Sigma-Aldrich, St. Louis, MO, USA; #S1382).

MCF7 and HEPG2 cells (ATCC HTB-22, HB-8065, respectively) were cultured in DMEM medium (ThermoFisher, Waltham, MA, USA; #11966025) containing 10% FBS and 1% penicillin/streptomycin for 18 h (75% confluence). Sodium selenite was added to the medium at final concentrations of both 0.5 µM and 1 µM, respectively, for an additional 24 h during the cells’ growth phase. The cells were collected at 90% confluence and subjected for Western blot analysis. It is important to mention that MCF7 cells were isolated from a female host and HEPG2 cells were isolated from a male host. In this manner, we collected data to support that there are no sex-discrepancies in selenite’s inhibitory action of Notch signaling.

### 2.2. Animals

Eight cage- and age-matched male mice (Jackson Lab, Bar Harbor, ME, USA; C57BL/6J) (average body weight 25 g) were used in this study. Four male mice at random were assigned to the control and selenite treated groups (*n* = 4/group). A final concentration of 2.5 mg/kg sodium selenite was delivered via intraperitoneal (IP) injection. Prior to injection, the mice were fasted for four hours, and then for six hours after injection. After the animals were euthanized, both the liver and kidney tissues were isolated and processed for Western blot (discussed below). All animal work has been approved through Oakland University’s IACUC.

### 2.3. Bioinformatics

Total RNA was isolated from primary cultured hepatocytes as described in Section 2.1. The total RNA was submitted and quantified for RNA-seq and library preparation (Genewiz, South Plainfield, NJ, USA). The Illumina HiSeq2000 was picked for all subsequent sequencing. Two-tailed RNA-seq was performed at 500 million counts with 150 bp read lengths. All quality control analyses, read alignments, and read countings were performed on a high-performance computing environment on a Linux operating system hosted by The Institute for Cyber-Enabled Research at Michigan State University. Reads were subjected to differential expression assays using hisat2 v.2.1.0 for read alignment, HTSeq-Count v.0.11.2 for feature counting, and DESeq2 v.1.28.1 for count normalization and scaling. To normalize read counts for inter-sample comparisons, DESeq2’s standard pipeline was used to estimate size factors for read scaling using the available R package. The DESeq2 function was used to compute log fold-changes and *p*-values for each feature. Multiple tools for pathway analysis, including Wikipathway, KEGG, Panther and Ingenuity Pathway Analysis (IPA) were used to further analyze gene sets. *p*-values at 10^−6^ were used for the critical significance threshold. Currently, this RNA-seq data is available on the GEO database (NCBI).

### 2.4. Quantitative Real-Time PCR

Total RNA was extracted from primary hepatocytes as well as immortalized cancer cell lines, MCF7 and HEPG2. The Qiagen Miniprep Kit (Qiagen, Hilden, Germany; #27104) was utilized for primary hepatocyte extraction, and the ZYMO Direct-zol RNA MiniPrep Plus kit (ZYMO Research, Irvine, CA, USA; #R2070) was used for the two cancer lines. The surfaces were first cleaned and sprayed with RNaseZap (ThermoFisher, Waltham, MA, USA; # AM9780) to eliminate any endogenous RNAse activity. Then, 900 μL of TriZol reagent was added to each well in the 6-well plate. Cells were aspirated and then processed using both kits, respectively. Once total RNA was extracted, it was quantified and assessed for purity using a NanoDrop2000. Reverse transcriptase was then performed using the M-MuLV Reverse Transcriptase Kit (NEB, Ipswich, MA, USA; #M0253S). To produce 20 μL of cDNA PCR product, 1 μg of template RNA was used. After cDNA synthesis, Luna Universal qPCR Master Mix (NEB, Ipswich, MA, USA; # M3003S) was used to make a 20 μL reaction. Primers for these reactions can be found in Table 1. The reaction was carried out in a CFX Connect Real-Time PCR Detection System (BIORAD, Hercules, CA, USA) for 40 cycles. Extension was carried out at 60 °C for 30 s before plate reading.

### 2.5. Western Blot and ICP-MS

MCF7 and HEPG2 cell lysates were collected from 6-well plates using a cell scraper and 150 μL of RIPA buffer. The lysates were placed in ice and subjected to two rounds of freeze–thaw membrane disruption. The lysate was then sonicated three times on ice until it became clear. The lysate was then spun down at 12,000× *g* for 20 min and protein was collected and quantified using BCA assay. Then, 10 μg of sample was loaded per well and separated using an SDS gel. Liver and kidney tissues were harvested immediately after euthanasia and placed in liquid nitrogen. Tissues were then recovered from liquid nitrogen and 30 mg was weighed and homogenized in RIPA buffer using a bullet homogenizer. The homogenate was agitated for 30 min at 4 °C on a microcentrifuge rotator and then spun down at 12,000× *g* at 4 °C. Protein was collected and a BCA assay was performed to quantify protein homogenate. Subsequently, 30 μg of protein was loaded into wells and separated using 8% SDS gels. Proteins were transferred onto PVDF membranes for two hours in an ice bath at 70 V. Membranes were blocked in 5% nonfat dry milk for 1 h at room temperature. Primary antibodies were diluted in PBST and incubated overnight at 4 °C with gentle agitation. Secondary antibody was diluted in 5% milk blocking buffer and incubated at room temperature for 1 h. Membranes were washed and imaged using ECL. The antibodies used were anti-Notch1 1:2000 (Cell Signaling, Danvers, MA, USA; #3608), anti-Notch1 1:2000 (Cell Signaling, Danvers, MA, USA; #4380), anti-alpha tubulin 1:5000 (Proteintech, Rosemont, IL, USA; 11224-1-AP), and anti-GAPDH 1:10,000 (Proteintech, Rosemont, IL, USA; 10494-1-AP). Anti-Notch1 (#3608) was preferred in cell blots and resulted in consistent bands at the indicated size. However, anti-Notch1 (#4380) was used in tissue blots yielding proper bands unlike #3608. For unexplained reasons, #3608 produced many bands at varying sizes in tissues never showing the product listed band.

Selenium in tissues was quantified by inductively-coupled-plasma mass spectrophotometry (ICP-MS), as described previously [13]. In brief, tissues were weighed at 50 mg and digested in 70% nitric acid at 70 °C until completely digested. Samples were then diluted in HPLC grade water and centrifuged to remove undigested tissue particulates. The supernatant was collected and transferred to a new tube. Quantification of elemental selenium was performed by ICP-MS (Nexion 300; Perkin Elmer, Waltham, MA, USA). Results were calculated at the final unit of ng/mg tissue.

## 3. Results

### 3.1. Transcriptomics Assays Identified Delta–Notch Signaling as a Significant Selenite Regulated Target

**Figure 1:**
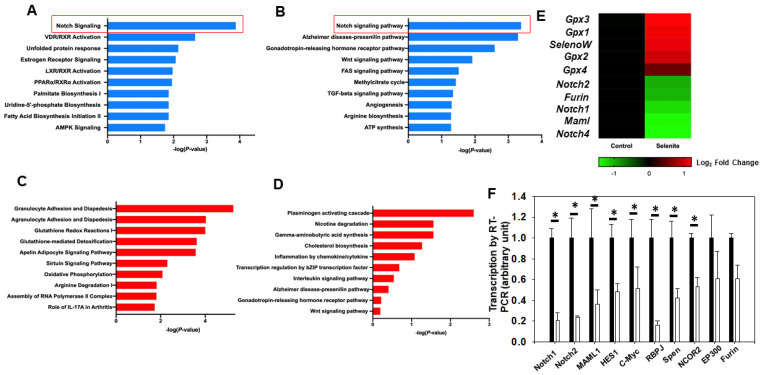
Transcriptomics analysis disclosed selenite downregulation of the Delta–Notch pathway in primary isolated hepatocytes. (**A**) Inuity Pathway Analysis showed pathways downregulated following selenite treatment. (**B**) GoPathway showed major pathways downregulated following selenite treatment. (**C**) Ingenuity pathway analysis showed pathways upregulated following selenite treatment. (**D**) GoPathway showed major pathways downregulated following selenite treatment. (**E**) Heat map plotting of differentially-expressed genes involving selenoproteins (*Gpx1*, *Gpx3*, *Gpx4* and *Selenow*) and Notch signaling pathways (*Notch1*, *Notch2*, *Notch4*, *Furin* and *Maml1*). Data were normalized by a regularized log transformation. (**F**) Quantitative RT-PCR results showed the decrease in Notch1 signaling members, normalized by alpha tubulin (*n* = 3). Ingenuity pathway analysis of the RNA-seq data was used to predict singling pathway activity (using a cut-off of *p* < 0.05 and the top 500 differentially expressed genes). * *p* < 0.05.

To detect potential downstream molecules perturbed by selenite, we treated primary hepatocytes with supranutritional selenite at a previously determined concentration, 2 µM [27]. Notably, liver cells in general show high tolerance to high concentrations of selenite. We have observed healthy growth in liver cells at concentrations above 30 µM of supplemented sodium selenite (preliminary data in our lab). We then compared the gene expression profiles between Se-treated hepatocytes and non-treated controls. RNA sequencing analysis identified 1259 differentially expressed genes (q < 0.05, |log_2_ fold change| > 0.5). Pathway analysis using ingenuity pathway analysis (IPA) and GoPathway (Gene Ontology Resource) identified that multiple pathways were significantly downregulated (Figure 1A,B) and upregulated after selenite treatment (Figure 1C,D). Consistent with its anti-oxidation function, selenite stimulates pathways including glutathione redox reactions (Figure 1E) and increases the expression of selenoproteins *Gpx1–4* and *Selenow* (selenoprotein W, Figure 1E). These LSKO cells have decreased *Zip8* expression; therefore, as well as being cultured in serum-free medium, the control condition is “selenium-deficient”, and the comparison was performed between a Se-deficiency condition and a selenite-supplemented condition.

Among those most significantly deregulated pathways (Figure 1A,B), we found that selenite downregulates Notch signaling and an enrichment of Notch signaling genes were pooled out, including *Notch1*, *Notch2*, *Notch4*, *Maml1* and *Furin*. QRT-PCR further confirmed the decrease in seven genes involved within Notch signaling, those being: *Notch1*, *Notch2*, *Maml1*, *Hes1*, *c-Myc*, *Rbpj*, *Spen*, and *Ncor2* (primers for qRT-PCR are listed in Table 1. All seven genes were down regulated by 40–70% with statistical significance (*p* < 0.05, Figure 1F). The results from RNA-seq and QRT-PCR are largely consistent with one another, leading us to believe that a connection truly exists between selenite and Notch signaling.

### 3.2. Selenite Inhibits Notch1 Expression in Cancer Cell Lines

Selenite, in prior studies, has displayed anti-proliferative properties in cancer cells. Therefore, we chose to study Notch expression in a breast adenocarcinoma cell line, MCF7, as well as a liver carcinoma cell line, HEPG2. We adopted two physiological selenite concentrations, 0.5 µM and 1.0 µM, for cell treatment.

**Figure 2:**
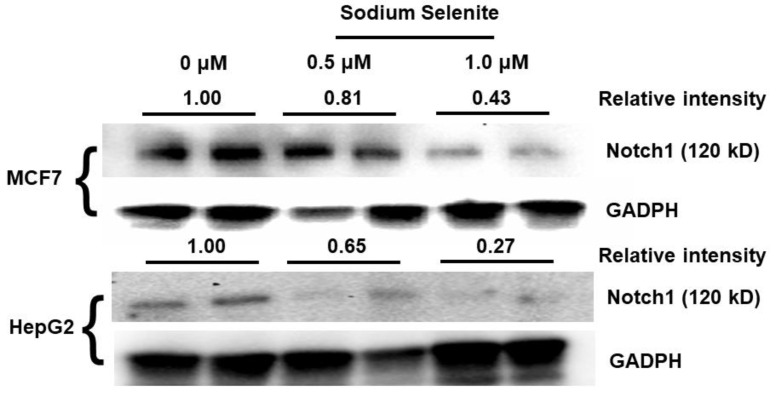
Selenite inhibits Notch1 protein expression in mammalian cancer cell lines MCF7 and HEPG2. Selenite was added to culture at indicated concentrations overnight, and cells were collected for the Western blot. Western blot of treated MCF7 shows a decrease in Notch1 intracellular/transmembrane region (NTM) expression at 1.0 μM after 24 h in MCF7 cultures (*n* = 2). HEPG2 at both 0.5 μM and 1.0 μM showed a decrease in Notch1 (NTM) expression after 24 h (*n* = 2). GAPDH was utilized as the loading control for all cell sample data. Averages of replicates in their relative expression (Notch1 versus GADPH) are indicated in the figure for easy comparison.

As shown in Figure 2, selenite significantly inhibited Notch1 in a concentration-dependent manner. Notch1 protein expression, quantified by Western blot using commercial antibody, were significantly decreased at 1.0 µM in MCF7; however, at 0.5 µM, less change was observed. Differential Notch1 production was detected in HEPG2 at both selenite concentrations, showing that selenite behaves conditionally based on cell type. The relative expressions of Notch1 versus GAPDH, quantified by band intensity, are labeled as average on the figure for easy comparison.

Along with the initial bioinformatic results, data from these MCF7 and HEPG2 cancer cells further confirmed downregulation of Notch1 by selenite. Whether selenite downregulates other Notch-family receptors and targets requires further investigation.

### 3.3. Selenite Inhibits Notch1 Expression in Mouse Liver and Kidneys

**Figure 3:**
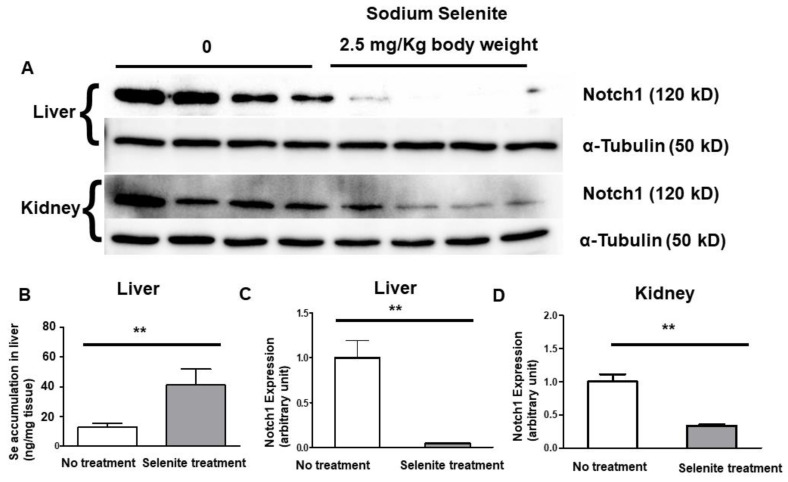
Selenite inhibits Notch1 expression in mouse liver and kidney. Sodium selenite was administrated via intraperitoneal (IP) injection at a final dosage of 2.5 mg/kg for 6 h treatment. After treatment, liver and kidney tissues were collected for Notch quantification by Western blot. (**A**) Western blot of Notch1 intracellular/transmembrane region (NTM) in liver and kidney tissue homogenates. Alpha-tubulin was preferred as the loading control for all in vivo tissue samples. (**B**) Quantification of selenium in treated and non-treated mouse livers using ICP-MS to verify selenium accumulation and injection quality. *n* = 4 for both untreated and selenite treated wild-type mice. Western blot quantification of liver (**C**) and kidney (**D**) shows a significant decrease in Notch1 protein expression after receiving sodium selenite treatment (** *p* < 0.01).

To further understand selenite’s inhibitory capacity in Notch signaling, we tested the protein expression of Notch1 in intact animals following selenite treatment. Sodium selenite was administered in mice at a bolus dosage of 2.5 mg/kg, a sub-lethal dose. Six hours after injection, mice were sacrificed and liver and kidney tissues, both known for high selenium retention, were excised. Elevated selenium levels in the liver tissues showed that selenite was not eliminated within the six-hour treatment period (Figure 3B). Our Western blot showed that Notch1 expression was considerably downregulated (Figure 3A) in both tissues. A significant decrease of 90% was observed in the liver (Figure 3C), and 70% (Figure 3D) in the kidney.

We noticed that the Notch1 antibody (Cell Signaling, Danvers, MA, USA; #3608), when applied in tissue lysate, did not probe like it did for cell lysates at the correct size. The antibody probed a band at lower molecular weight, around 80 kD, which is lower than that normally expected at 120 kD (surprisingly, the 80 kD band also showed a significant decrease in selenite-treated samples), which is possibly a reduced Notch1 form. Another Notch1 antibody (Cell Signaling, Danvers, MA, USA; #4380) was then adopted, ultimately displaying a single band at the predicted size of 120 kD.

In tissue lysate, we also attempted to study the expression of Notch signaling targets Maml1, c-Myc, Cleaved Notch1 and Hes1 by Western blot; however, the commercial antibodies (Cell Signaling, Danvers, MA, USA; #12166, #5605, #4147, #11988, respectively), did not show proper band sizes and were non-specific. We concluded, through various trials and troubleshoots, that these antibodies proved ineffective in probing protein expression. Therefore, we are not confident in making an accurate conclusion regarding selenite’s downstream effect on these targets in tissue lysates.

## 4. Discussion

### 4.1. Possible Mechanisms of Selenite Regulated Notch Signaling

In the ZIP8 knockout hepatocytes cultured in a Se-deficiency media, transcriptomics showed that supplementation of selenite increased various selenoprotein expressions. Such elevations, however, were not observed in cells being supplemented with supra-nutritional selenite under normal media conditions [28]. By using pathway analyses, including KEGG, Panther and IPA, Delta–Notch signaling appears to be one of the most significant selenite-regulated targets (*p* < 10^−6^).

Selenite inhibition of Notch signaling was further verified in cultured primary and cancerous hepatocytes, breast cancer cells and liver and kidney tissues. Downregulation of Notch signaling includes *Notch1* and associated genes including its modifiers and targets *Maml1*, *Hes1*, *c-Myc*, etc., in selenite treated primary hepatocytes. Downregulation of Notch1 was further observed in MCF7 and HEPG2 cancer cells as well as in both liver and kidney tissues, two organs known for high selenium retention [29,30]. Selenite’s inhibitory role of liver Notch1 was observed to be greater in comparison to kidney tissues. A plausible explanation could be the liver’s requirement to neutralize oxidative species during nutrient metabolism and ATP production. Biological selenium has endogenous activity to behave as an antioxidant [31], which could explain why the liver’s requirement for selenium is higher compared to that of the kidneys. The higher selenium content in the liver after selenite IP injection supports this hypothesis. However, the mechanism for the difference in selenium accumulation has yet to be studied in a laboratory setting.

The MCF7 line was derived from female tissue, the HEPG2 line from males, and all our animals in these experiments were males; therefore, our results indicate that selenite inhibition of Notch signaling could be a ubiquitous event in cells and tissues, regardless of sex.

Notch signal transduction occurs upon cell–cell interaction with five common canonical Notch ligands: Delta-like (Dll) 1, 3, 4 and Jagged 1 and 2 [32]. Once the connection between the Notch ligand and receptor has occurred, cleavage by post-translational enzymes such as Furin, ADAM and γ-secretase allows the signaling cascade to proceed. The cleaved portion of the Notch receptor, NICD, can then translocate into the nucleus where it forms a complex with other proteins to act as a transcription factor. Glycosylation and modifications through ubiquitin attachment can regulate pre-Notch assembly, reducing its presence at the cell surface [32].

While the detailed mechanism needs more elucidation, based on current findings and understandings of selenite function, we propose several plausible mechanisms that could explain how such inhibition may occur during transcription and/or post-translation processing.

One plausible mechanism of selenite inhibition is via selenite’s anti-inflammatory effect. Transcriptome studies disclosed that selenite represses multiple interleukin secretions in leukocytes [33], as well as inhibiting NF-κB transcriptional activities in cells [34]. NF-κB, a master inflammation transcriptional factor, controls hundreds of downstream genes including Notch. We believe that the interaction with the NF-κB pathway is the most relevant crosstalk described for selenite inhibition of Notch and related genes.

Numerous reports have investigated the complicated mechanisms between Notch and NF-κB in diverse experimental models [35,36]. Evidence showed that the Notch and NF-κB pathways positively regulate one another at a transcriptional level; thus, it is plausible that the direct inhibition of NF-κB will sequentially decrease Notch1 and related gene transcription. The interaction between Notch and NF-κB was also found to be physical between N-terminal Notch1 and NF-κB p50 subunits [37].

One concern regarding this hypothesis is that NF-κB was not activated in hepatocytes and normal tissues used in this study; whether basal NF-κB expression also effects Notch signaling still needs to be determined. Future studies involving cytokine treatment to induce NF-κB activation in relative cells could further clarify this mechanism.

A second mechanism, which may explain selenite inhibition, is via multiple zinc-containing proteinases. Selenite inhibits pathways and molecules involved in cell migration, epithelial to mesenchymal transition (EMT), and fibrosis. Selenium competitively binds with thiols at binding sites normally associated with zinc in many Zn-dependent proteinases, such as ADAMs and MMPs, exerting inhibitory effects [38]. These proteinases are widely involved in Notch processing and activation; therefore, their activities regulate Notch signaling at a post-translational level.

One of the proteinases, γ-secretase, is a plausible selenite target. The γ-secretase complex cleaves Notch1 and releases the intracellular domain (NICD) into the cytosol. The cleaved Notch then proceeds into the nucleus to be activated and contains subunits of *Psen*, *Aph1* and *Pen*. Our RNA-seq data supports the moderate decrease in *Psen1* and *Psen2* (data not shown), showing potential evidence for this hypothesis. Additional evidence for this hypothesis can be found in previous studies showing that selenite inhibits γ-secretase activity through the activation of ERK in the range of 2–6 µM in kidney cells, HEK293T [39].

In addition, ubiquitination also regulates the Notch pathway through the proteasome degradation pathway. The involvement of E1, ubiquitin-activating enzyme; E2, ubiquitin-conjugating enzyme; and E3, ubiquitin ligase, are essential for maintaining proper Notch protein expression [32]. Although selenite was found to be indirectly involved with ubiquitination in mammals, there is currently little evidence to explain this interaction.

We discussed that selenite function works in a dose-dependent manner. This study adopted a dose of 2 µM selenite for primary hepatocytes, 0.5 µM and 1 µM for cancer cells, and 2.5 mg/kg for mice. Under these concentrations, selenite strictly exerts inhibitory properties.

Proposed selenite inhibition of Notch signaling was outlined in the graphical abstract. Inhibition via transcription (NF-κB) and protein processing (proteinase or γ-secretase gamma secretase) were predicted due to their capacity to regulate Notch1 and Notch1 related diseases. Although we only studied Notch1, other Notch family members may be subject to similar inhibition by selenite, under different cellular and tissue conditions.

### 4.2. Clinical Significance of Selenite Inhibition via Notch Signaling

Selenite inhibition of Notch signaling shows promising clinical significance. One potential application of selenite–Notch inhibition is in cancer treatment. Notch is one of the most potent stem cell-promoting pathways, in conjunction with the Hedgehog and Wnt pathways, making it a highly relevant target for cancer therapies [22,40,41]. In general, Notch promotes cell survival and angiogenesis leading to overall treatment resistance in numerous cancers, deeming it an oncogenic signal pathway. It cross-talks with several other critical oncogenic pathways including EGFR and Ras [42,43,44]. Inhibition by Notch allows for one master target to regulate numerous signaling pathways involved in cancer development and progression. Currently, blocking γ-secretase is the preferred strategy to regulate Notch as other new inhibitory molecules and targets emerge.

Selenite, again, is well-known for its affinity and toxicity towards cancer cells [5]. This could be explained by the high levels of glutathione (GSH) in the cancer tissue environment which facilitates selenite reduction to its free-permeable and toxic form, selenide (HSe^−^). Selenide may then target various pathways and exert its anti-cancer function. Selenite has also been found to interact with thioredoxin reductase (TR/Trx1), a selenoprotein responsible for redox-sensitive pathways such as cell proliferation and DNA replication. Selenite was shown to inhibit Trx1 and allow chemotherapeutic drugs such as acylfulvene or illudin S to be more efficient [45]. This validates selenite as a cancer therapy adjuvant. We suggest in this study that selenite inhibition of Notch offers a new mechanism for cancer toxicity.

Targeting Notch also shows potential treatment benefits for fibrogenesis. As mounting evidence shows, both animal models and human studies have indicated that Notch signaling is involved in fibrotic pathogenesis [46]. Selenite suppression of Notch1 may suggest a route of regulation for multiple fibrogenesis mechanisms such as TGF-β, MMP9, etc.

The selenite–Notch inhibition may also have some potential use in the therapy of neurodegenerative disorders, such as Alzheimer’s disease (AD), via the inhibition of γ-secretase [47]. Some longitudinal and cross-sectional studies show an association of selenium status and cognitive function [48,49].

To summarize this study as a whole, the novel identification of Notch regulation by inorganic selenite provides an initial look into a unique selenite driven mechanism. To understand selenium’s plausible clinical application in the treatment of cancers, AD, fibrosis and other Notch-related pathologies, future studies will surely build upon this preliminary discovery.

## Figures and Tables

**Table 1:** Primers for qRT-PCR.

Gene Names	Primer Sequences (5′-3′)	Protein Function
*Notch1*	Forward: GCC GCA AGA GGC TTG AGA T Reverse: GGA GTC CTG GCA TCG TTG G	Notch1 signaling
*Notch2*	Forward: CAG GAG GTG ATA GGC TCT AGG Reverse: GAA GCA CTG GTC TGA ATC TTG	Notch2 signaling
*HeS1*	Forward: ACA CCG GAC AAA CCA AAG AC Reverse: AAT GCC GGG AGC TAT CTT TC	Transcriptional Suppressor
*c-Myc*	Forward: AAC AAC CGC AAG TGC TCC AG Reverse: TCT CGT CGT TTC CTC AAT AAG TCC	Transcription factor for apoptosis and cell growth
*Manl1*	Forward: TCA CAA GCA AGA TGA TGA GCA CAG Reverse: GCA CGG AAG TCA CTC CAG CA	Transcriptional coactivator for Notch proteins
*Spen*	Forward: AGT TTC GGC CCT TGG ATG AG Reverse: GCA CCC CGT TCA CCT TCT TA	Hormone inducible transcriptional repressor
*Ep300*	Forward: TTC AGC CAA GCG GCC TAA AC Reverse: GTT CCA GGT CAA ACA GTG AAC CAA	Transcriptional coactivator protein
*Rbpj*	Forward: GCG TCC CAA AAC CCG GAT AA Reverse: GTG AGT CGT TTG GGT GGA GG	Transcriptional regulator for Notch-related proteins
*Furin*	Forward: CAT GAC TAC TCT GCT GAT GG Reverse: GAA CGA GAG TGA ACT TGG TC	Processes pre-Notch1 in the Golgi network
*Ncor2*	Forward: CGC ACA CCG GAT CCT AGA AG Reverse: TCC GCT TAA AGT ACA AGA TCA GCT T	Nuclear receptor corepressor that mediates transcriptional repression
*Alpha tubulin*	Forward: CTC GCC TTC TAA CCC GTT GCT Reverse: TCT TGT CAC TTG GCA TCT GGG	Housekeeping control

## Data Availability

The data discussed in this publication have been deposited in NCBI’s Gene Expression Omnibus (Liu et al. 2021) and are accessible through GEO Series accession number GSE167626 (https://www.ncbi.nlm.nih.gov/geo/query/acc.cgi?acc=GSE167626).

## Abbreviations

Se	Selenium
IPA	Ingenuity pathway analysis
GSH	Glutathione
ROS	Reactive oxidative species
AD	Alzheimer’s disease
EMT	Epithelial–mesenchymal transition
IP injection	Intraperitoneal injection
ICP-MS	Inductively-coupled-plasma mass spectrophotometry
